# Assessment of Thyroid Function in Patients With Alkaptonuria

**DOI:** 10.1001/jamanetworkopen.2020.1357

**Published:** 2020-03-23

**Authors:** Shirisha Avadhanula, Wendy J. Introne, Sungyoung Auh, Steven J. Soldin, Brian Stolze, Debra Regier, Carla Ciccone, Fady Hannah-Shmouni, Armando C. Filie, Kenneth D. Burman, Joanna Klubo-Gwiezdzinska

**Affiliations:** 1 National Institute of Diabetes and Digestive and Kidney Diseases, National Institutes of Health, Bethesda, Maryland; 2 National Institute of Child Health and Human Development, National Institutes of Health, Bethesda, Maryland; 3 National Human Genome Research Institute, National Institutes of Health, Bethesda, Maryland; 4 Department of Laboratory Medicine, National Institutes of Health, Bethesda, Maryland; 5 Children’s National Rare Disease Institute, Children’s National Medical Center, Washington, DC; 6 National Cancer Institute, National Institutes of Health, Bethesda, Maryland; 7 Endocrine Section, Medstar Washington Hospital Center, Washington, DC

## Abstract

**Importance:**

Alkaptonuria is an autosomal recessive disorder caused by pathogenic variants in the *HGD* gene. Deficiency of the HGD enzyme leads to tissue deposition of homogentisic acid (HGA), causing severe osteoarthropathies and cardiac valve degeneration. Although HGD is vital for the catabolism of tyrosine, which provides the basis for thyroid hormone synthesis, the prevalence of thyroid dysfunction in alkaptonuria is unknown.

**Objective:**

To assess thyroid structure and function in patients with alkaptonuria.

**Design, Setting, and Participants:**

A single-center cohort study was conducted in a tertiary referral center including patients with alkaptonuria followed up for a median of 93 (interquartile range, 48-150) months between February 1, 2000, and December 31, 2018. The alkaptonuria diagnosis was based on clinical presentation and elevated urine HGA levels. A total of 130 patients were considered for participation.

**Main Outcomes and Measures:**

Prevalence of thyroid dysfunction in adults with alkaptonuria compared with the general population. Thyrotropin and free thyroxine levels were measured by immunoassay and repeated in each patient a median of 3 (interquartile range, 2-22) times. Neck ultrasonographic scans were analyzed in a subset of participants. Logistic regression was used to test the association of thyroid dysfunction with age, sex, thyroid peroxidase (TPO) antibodies, serum tyrosine levels, and urine HGA levels.

**Results:**

Of the 130 patients, 5 were excluded owing to thyroidectomy as the cause of hypothyroidism. The study cohort consisted of 125 patients; the median age was 45 (interquartile range, 35-51) years. Most of the patients were men (72 [57.6%]). The prevalence of primary hyperthyroidism was 0.8% (1 of 125 patients), similar to 0.5% observed in the general population (difference, 0.003; 95% CI, −0.001 to 0.04; *P* = .88). The prevalence of primary hypothyroidism was 16.0% (20 of 125 patients), which is significantly higher than 3.7% reported in the general population (difference, 0.12; 95% CI, 0.10-0.24; *P* < .001). Women were more likely to have primary hypothyroidism than men (odds ratio, 10.99; 95% CI, 3.13-38.66; *P* < .001). Patients with TPO antibodies had a higher likelihood of primary hypothyroidism than those without TPO antibodies (odds ratio, 7.36; 95% CI, 1.89-28.62; *P* = .004). There was no significant difference in the prevalence of thyroid nodules between patients in this study (29 of 49 [59.2%]) vs the general population (68%) (difference, 0.088; 95% CI, −0.44 to 0.73; *P* = .20) or of cancer (7% vs 5%; difference, 0.01; 95% CI, −0.01 to 0.17; *P* = .86).

**Conclusions and Relevance:**

The high prevalence of primary hypothyroidism noted in patients with alkaptonuria in this study suggests that serial screening in this population should be considered and prioritized.

## Key Points

**Question:**

Is alkaptonuria associated with thyroid dysfunction?

**Findings:**

In this cohort study, 125 adults with alkaptonuria followed up for a median of 93 months were found to have a 16.0% prevalence of primary hypothyroidism. This prevalence was significantly higher than the 3.7% prevalence in the general population.

**Meaning:**

Results of this study suggest that screening for hypothyroidism should be considered in patients with alkaptonuria.

## Teaser

This cohort study examines thyroid function and structure in patients with alkaptonuria.

## Introduction

Alkaptonuria is a rare, autosomal recessive inherited metabolic disorder, with an estimated incidence rate in the US ranging from 1 in 250 000 to 1 in 1 000 000 live births.^1^ A significantly higher prevalence of alkaptonuria (1:19 000) has been observed in some regions, including the Dominican Republic and in northwestern Slovakia, likely owing to a founder effect.^2^ Alkaptonuria is caused by pathogenic variants in the *HGD* (OMIM *607474) gene, leading to deficiency of the HGD enzyme, which converts homogentisic acid (HGA) to maleylacetoacetic acid in the tyrosine degradation pathway (eFigure in the Supplement).^1^ In this disorder, excess HGA is excreted in the urine, leading to its darkening on exposure to air and consequent oxidation of HGA, producing a melaninlike product. However, the typical black urine disease is often not diagnosed early in life, as darkening of the urine may not occur for several hours after voiding and thus might not be recognized in a timely manner. Therefore, the clinical presentation of the disease often does not manifest until the fourth or fifth decade of life when chronic tissue accumulation of HGA leads to ochronosis (bluish-black pigmentation in connective tissue), early degenerative joint disease affecting mainly the spine and large joints, pigment deposition in cardiac valves leading to valvulopathy, as well as kidney and prostate stones.^1,3,4,5,6,7^

Treatment of alkaptonuria is supportive, including pain management, physiotherapy, and joint and aortic and/or mitral valve replacement surgery.^1,5^ In 1998, nitisinone, a potent inhibitor of para-hydroxyphenylpyruvate dioxygenase, the second enzyme in the tyrosine degradation pathway, was introduced for the management of alkaptonuria.^8^ Although a clinical trial using nitisinone in patients with alkaptonuria revealed a significant biochemical improvement with reduction of HGA levels in the urine by more than 95%, significant clinical improvement could not be proven.^9^

Homogentisic acid, the metabolic by-product that accumulates in alkaptonuria, has structural similarity to tyrosine. Because tyrosine is essential for thyroid hormone synthesis, we hypothesized that when HGA is present in higher amounts, it may compete with tyrosine for transport into the thyroid gland, affecting tyrosine levels locally in the tissue, or be inappropriately incorporated into thyroglobulin, leading to aberrant thyroid hormone production (eFigure in the Supplement). Therefore, the goal of this study was to assess thyroid function and structure in a large cohort of patients with alkaptonuria.

## Methods

We performed a retrospective cohort study of adults with alkaptonuria who were enrolled at the National Institutes of Health from February 1, 2000, to December 31, 2018. Participants were enrolled at a single center, the National Institutes of Health, and were from primarily North America and Europe. Alkaptonuria diagnosis was based on clinical, biochemical (elevated urine HGA level), and genetic evaluations. We included patients who had thyroid function tests (thyrotropin and free thyroxine) repeated at least twice and information on medical management of their thyroid disorder. Individuals with postsurgical hypothyroidism were excluded from the analysis. The study was approved by the National Institutes of Health Institutional Review Board. Written informed consent was obtained from all study participants. Participants did not receive financial compensation. This study followed the Strengthening the Reporting of Observational Studies in Epidemiology (STROBE) reporting guideline for cohort studies.

The primary end point of the study was to analyze the prevalence of thyroid dysfunction in patients with alkaptonuria and compare the prevalence with a standard US adult population used as a control sample.^10,11,12^ The secondary end point was to study the association of thyroid dysfunction with age, sex, and thyroid peroxidase (TPO) antibodies, as well as serum tyrosine and urine HGA levels. Demographic, clinical, and biochemical data were obtained from medical records and patient interviews. Detailed information regarding potential confounding variables was obtained, including comorbidities, use of medications potentially affecting thyroid function (eg, amiodarone, estrogens, and glucocorticoids), and use of medications potentially affecting measurements of thyroid hormones (eg, biotin).

Serum thyrotropin and free thyroxine levels were measured by immunoassay and were repeated in each patient a median of 3 (interquartile range [IQR], 2-22) times. The results of thyroid function tests were verified by liquid chromatography with tandem mass spectrometry at least once during follow-up. Overt hypothyroidism was defined as a thyrotropin level above the reference range (between 2010 and 2014: 0.40-4.00 mIU/L and between 2014 and 2018: 0.27-4.2 mIU/L) and a free thyroxine level below the reference range (between 2010 and 2014: 0.9-1.5 ng/dL and between 2014 and 2018: 0.9-1.7 ng/dL [to convert to picomoles per liter, multiply by 12.871]) on 2 tests or normalization of thyrotropin levels on weight-based levothyroxine replacement therapy. Subclinical hypothyroidism was defined as a thyrotropin level above the reference range with free thyroxine levels within the reference range on 2 tests.^12,13^ Overt hyperthyroidism was defined as decreased/suppressed thyrotropin levels with elevated free thyroxine levels, and subclinical hyperthyroidism was defined as thyrotropin levels below the lower limit of the reference range with free thyroxine levels within the reference range measured on at least 2 occasions.^14^ Thyroid peroxidase antibodies were measured by immunoassay (reference range, <10-35 IU/mL). The mean serum tyrosine concentration (reference range, 0.56-1.63 mg/dL [to convert to micromoles per liter, multiply by 55.19]) was measured by liquid chromatography with tandem mass spectrometry, repeated a median of 3 (range, 1-24) times in each patient. The mean urine HGA concentration (reference range, <11 mmol/mol creatinine [Cr]) was measured by gas chromatography–mass spectrometry in a 24-hour urine collection, repeated a median of 3 (IQR, 1-13) times in each patient. Seventeen of 125 patients (13.6%) with alkaptonuria were receiving nitisinone therapy between 2005 and 2009.

Neck ultrasonographic scans were analyzed in a subset of patients. Thyroid volume was assessed using the following formula: volume (milliliters) = 0.479 × depth × width × length.^15^

In addition, available formalin-fixed paraffin-embedded thyroid specimens from patients who underwent thyroidectomy (n = 4) were analyzed with Schmorl stain to assess for any alkaptonuria-related pigment accumulation in the thyroid gland. A thyroid tissue specimen from a patient without alkaptonuria served as a negative control and a pigment deposition in the skin of a patient without alkaptonuria served as a positive control. The Schmorl method uses the reduction of ferricyanide to ferrocyanide, which, in the presence of ferric ions, forms a Prussian blue stain (ie, stains pigment blue-green), signifying HGA accumulation.^16^

### Statistical Analysis

Baseline clinical characteristics of the study population were summarized using means (SDs) or medians with IQRs for continuous variables and proportions for categorical outcomes. Two-sample *t* test or Mann-Whitney test was used for group comparison of continuous variables. The χ^2^ or Fisher exact χ^2^ test was used for group comparison of categorical outcomes. To compare the study population with the general population for a binary outcome, such as primary hyperthyroidism, hypothyroidism, thyroid nodules and cancer, TPO antibodies, and categorized age and sex, we obtained Clopper-Pearson exact 95% CIs, and the Fisher exact test for 1-sample proportion was used with a reference proportion from the general population.

Simple logistic regression was performed to test the association of hypothyroidism with known clinically relevant variables, such as age, sex, and presence of TPO antibodies, as well as serum tyrosine and urine HGA levels. Estimated odds ratios (ORs) with corresponding 95% CIs are reported. All analyses were 2-tailed tests based on α = .05 and were conducted using SAS statistical software, version 9.4 (SAS Institute Inc).

## Results

**Figure 1.**
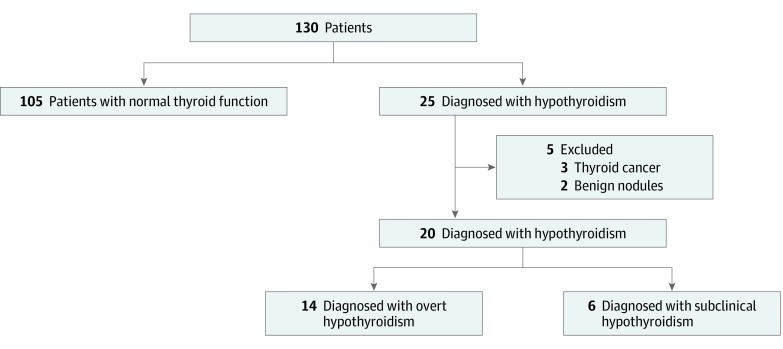
Flowchart of the Study Cohort

Among 130 adults with alkaptonuria, 5 were excluded owing to a history of postsurgical hypothyroidism (Figure 1). The final study cohort consisted of 125 patients with alkaptonuria (53 women [42.4%]), with a median age at first thyrotropin measurement of 45 (IQR, 35-51) years, who were followed up for a median of 93 (IQR, 48-150) months. One patient (0.8%) was diagnosed with primary hyperthyroidism due to Graves disease, initially manifesting as subclinical hyperthyroidism. The prevalence of hyperthyroidism was similar to the 0.5% prevalence observed in the general population (difference, 0.003; 95% CI, −0.001 to 0.04; *P* = .88).^10^ The reference general population was derived from a National Health and Nutrition Examination Survey study aimed at screening of the general population for thyroid disorders,^10^ and the demographics of the reference population were slightly different compared with the studied cohort. The age was comparable between the 2 populations; the proportion of patients aged 12 to 49 years in the National Health and Nutrition Examination Survey study was 68.5% (3010 of 4392) and aged 50 years of older was 31.5% (1382 of 4392), compared with the study cohort, with proportions of patients younger than 50 years of 69.6% (87 of 125; difference, 0.381; 95% CI, −0.61 to 0.78; *P* = .87) and aged 50 years or older, 30.4% (38 of 125; difference, 0.011; 95% CI, −0.22 to 0.39; *P* = .88). The 52.8% (2320 of 4392) prevalence of women in the National Health and Nutrition Examination Survey study was higher compared with the prevalence of 42.4% observed in this cohort (difference, 0.104; 95% CI, 0.34-0.52; *P* = .03).

Twenty patients were diagnosed with primary hypothyroidism (Figure 1). The prevalence of primary hypothyroidism was 16.0% (20 of 125) in the alkaptonuria cohort, which is significantly higher than 3.7% as reported in the general population by the National Health and Nutrition Examination Survey study (difference, 0.12; 95% CI, 0.10-0.24; *P* < .001).^10^ In this cohort, the median age at diagnosis of hypothyroidism was 51 (IQR, 48.25-60.25) years; the youngest age at diagnosis of hypothyroidism was 8 years. Twelve patients were found to harbor a diagnosis of hypothyroidism preceding study enrollment; in 8 patients, hypothyroidism had been detected during the follow-up period, accounting for an incidence of 6.4% (8 of 125) over 93 (IQR, 48-150) months.

Among patients with hypothyroidism, 14 of 125 patients (11.2%) were diagnosed with overt hypothyroidism; this rate was significantly higher than 0.3% observed in the general population (difference, 0.109; 95% CI, 0.06-0.18; *P* < .001).^10^ The patients with overt hypothyroidism were treated with a median levothyroxine dose of 100 μg (IQR, 78-119 μg), orally daily, which was equal to a median of 1.4 μg/kg of body weight (IQR, 1.1-1.6 μg/kg) and resulted in median thyrotropin levels (3.42; IQR, 2.08-4.47 mIU/L) within the reference range (Table 1). Thyrotropin levels obtained before the initiation of levothyroxine therapy were not available for analysis. Six of 125 patients (4.8%) were diagnosed with subclinical hypothyroidism and were not treated with thyroid hormones. The median thyrotropin level in patients with subclinical hypothyroidism was 5.7 mIU/L (IQR, 4.1-6.1 mIU/L).

**Table 1.** Baseline Characteristics of the Study Group

Variable	Median (IQR)			*P* value
	Study cohort (N = 125)	Hypothyroidism (n = 20)	Euthyroidism (n = 105)	
Age, y	45 (35-51)	49 (42-57)	44 (35-50)	.07
Women, No. (%)	53 (42.4)	14 (70.0)	39 (37.1)	.005
Weight, kg	77.6 (66.8-88.1)	68.6 (65.5-77.2)	73.2 (72.2-88.7)	.14
Elevated TPO antibodies, No./total No. (%)^a^	14/63 (22.2)	9/18 (50)	5/45 (11.1)	<.001
Serum tyrosine, mg/dL	1.07 (0.88-1.47)	1.20 (0.87-5.02)	1.05 (0.88-1.22)	.17
Urine HGA, mmoL/mol Cr	2030 (1141-3108)	2229 (1338-2991)	2039 (1144-3163)	.44
Thyrotropin, mIU/L^b^	1.66 (1.16-2.49)	3.72 (2.43-5.11)	1.52 (1.1-2.07)	<.001
Free thyroxine, ng/dL	1.21 (1.09-1.35)	1.2 (1.1-1.29)	1.21 (1.04-1.34)	.79
Thyroid volume measured by ultrasonography, mL	9.7 (7.5-12.3)	7 (3.9-13)	10.3 (7.8-12.2)	.12
Nitisinone, No. (%)	19 (15.2)	4 (20)	15 (14.3)	.51
Levothyroxine, No.	14	14	0	NA
Medications affecting thyroid function, No.				
Amiodarone^c^	1	0	1	NA
Estrogens	11	3	8	.29
Corticosteroids^d^	4	0	4	NA

Abbreviations: Cr, creatinine; HGA, homogentisic acid; IQR, interquartile range; NA, not applicable; TPO, thyroid peroxidase.

SI conversion factors: To convert free thyroxine to picomoles per liter, multiply by 12.871; tyrosine to micromoles per liter, multiply by 55.19.

^a^ Measurement of TPO antibodies performed for 63 patients.

^b^ Patients with overt hypothyroidism (n = 14) were treated with levothyroxine; the median thyrotropin level during levothyroxine therapy was 3.42 mIU/L (IQR, 2.08-4.47 mIU/L). Six patients with subclinical hypothyroidism were not treated with levothyroxine and were characterized by a median thyrotropin level of 5.7 mIU/L (IQR,4.1-6.1 mIU/L).

^c^ Two patients were treated with amiodarone; 1 of these patients underwent thyroidectomy for Hurthle cell thyroid cancer and was excluded from the study cohort.

^d^ Five patients were treated with corticosteroids; 1 of these patients was excluded from analysis because hypothyroidism was secondary to thyroidectomy for papillary thyroid cancer.

An analysis of comorbid conditions and therapy with medications known to potentially affect thyroid function was performed (Table 1). Two patients were treated with amiodarone. One of these patients underwent thyroidectomy for Hurthle cell thyroid cancer and was excluded from the study cohort; the remaining patient was euthyroid. Five patients were treated with corticosteroids; 1 of these patients was excluded from analysis because hypothyroidism was secondary to thyroidectomy performed for papillary thyroid cancer. All of the remaining 4 patients treated with corticosteroids were euthyroid. Eleven patients were treated with estrogens. There was no significant difference in the prevalence of estrogen therapy in patients with hypothyroidism (3 of 20 [15.0%]) compared with the euthyroid subset of the study cohort (8 of 105 [7.6%]) (*P* = .29).

The median age at enrollment of patients with hypothyroidism was not significantly different than that of individuals with normal thyroid function who had alkaptonuria (49 [42-57] vs 44 [35-50] years; *P* = .07) (Table 1). A significantly higher proportion of women was noted in the hypothyroid group (14 of 20 [70.0%]) compared with the euthyroid group (39 of 105 [37.1%]) (*P* = .005). Elevated levels of TPO antibodies were found in 9 of 18 patients (50.0%) with hypothyroidism vs 5 of 45 patients (11.1%) with normal thyroid function (*P* < .001). The median thyroid volume in the hypothyroid population was 7 mL (IQR, 3.9-13.6 mL), which was not significantly different from that of the euthyroid group (10.3; IQR, 7.8-12.2 mL) (*P* = .12). There were no significant differences between patients with alkaptonuria who were hypothyroid vs those who were euthyroid in serum tyrosine levels (1.20 [IQR, 0.87-5.02] mg/dL vs 1.05 [IQR, 0.88-1.22] mg/dL; *P* = .17) or urine HGA levels (2229 [IQR, 1338-2991] mmol/mol Cr vs 2039 [IQR, 1144-3163] mmol/mol Cr; *P* = .44) (Table 1).

Because 17 patients were transiently treated with nitisinone, which led to a short-term elevation in tyrosine levels and decrease in HGA levels, a separate analysis was performed for these individuals (eTable in the Supplement). Four patients in the hypothyroid group were treated with nitisinone, resulting in tyrosine levels of 11.21 mg/dL (IQR, 8.16-16.58 mg/dL) and HGA levels of 114 mmol/mol Cr (IQR, 81.3-140.6 mmol/mol Cr), which were not significantly different than the levels in the 13 patients who were euthyroid who, while receiving nitisinone, had median tyrosine levels of 13.60 mg/dL (IQR, 10.90-14.30 mg/dL; *P* = .98) and median HGA concentrations of 84 mmol/mol Cr (IQR, 67.4-117.4 mmol/mol Cr; *P* = .61) (eTable in the Supplement). All patients with hypothyroidism treated with nitisinone had hypothyroidism diagnosed before starting nitisinone therapy. Among patients with hypothyroidism not treated with nitisinone, the median tyrosine level was 1.02 mg/dL (IQR, 0.84-1.17 mg/dL) and the median HGA level was 2584.5 mmol/mol Cr (IQR, 1545.9-2962.7 mmol/mol Cr), which was not significantly different compared with patients who were euthyroid and had a median tyrosine level of 0.99 mg/dL (IQR, 0.85-1.10 mg/dL; *P* = .90) and a median HGA concentration of 2439.7 mmol/mol Cr (IQR, 1501-3221.4 mmol/mol Cr; *P* = .67) (eTable in the Supplement).

A simple logistic regression analysis revealed that women were 11 times more likely to harbor a diagnosis of primary hypothyroidism (OR, 10.99; 95% CI, 3.13-38.7; *P* < .001), while patients with TPO antibodies had a 7 times higher likelihood of primary hypothyroidism than their TPO antibody–negative counterparts (OR, 7.36; 95% CI, 1.89-28.6; *P* = .004) (Table 2). There was no association between primary hypothyroidism and age, serum tyrosine level, or urine HGA level (Table 2). Among patients with hypothyroidism and alkaptonuria, the prevalence of patients with positive TPO antibodies of 50.0% was significantly lower than the reported 82.3% prevalence in the general hypothyroid population (difference, 0.323; 95% CI, 0.26-0.74; *P* = .004).^17^

**Table 2.** Association Between Clinically Relevant Variables Using Logistic Regression Model

Variable	Odds ratio (95% CI)	*P* value
Female vs male^a^	10.99 (3.13-38.66)	<.001
Presence of TPO antibodies^a^	7.36 (1.89-28.62)	.004
Age	1.03 (0.98-1.08)	.20
Serum tyrosine level	1.00 (0.99-1.02)	.72
Urine HGA level	1.00 (0.99-1.00)	.62

Abbreviations: HGA, homogentisic acid; TPO, thyroid peroxidase.

^a^ Female sex and presence of TPO antibodies were associated with higher likelihood of hypothyroidism.

To further determine the clinical characteristics of patients with TPO-negative vs TPO-positive primary hypothyroidism, we performed a subgroup analysis. The patients with alkaptonuria and TPO antibody–negative hypothyroidism tended to be older than hypothyroid TPO antibody–positive patients (median, 53 [IQR, 47-58] years vs median, 42 [IQR, 17-57] years; *P* = .10) and had a smaller thyroid volume (median, 4 [IQR, 3.7-5.6] mL vs median, 14 [IQR, 13.6-14.2] mL; *P* = .01). There was no significant difference between the TPO-negative vs TPO-positive primary hypothyroidism groups in sex distribution (8 of 11 [73%] vs 6 of 9 [67%], *P* = .44), tyrosine levels (median, 1.14 [IQR, 0.91-1.23] mg/dL vs median, 5.02 [IQR, 1.41-8.77] mg/dL; *P* = .61), or urine HGA levels (median, 2044 [IQR, 988-2865] mmol/mol Cr vs median, 1732 [IQR, 1481-2967] mmol/mol Cr; *P* = .95) (Table 3).

**Table 3.** Comparison Between TPO Antibody–Positive and TPO Antibody–Negative Hypothyroidism in Patients With Alkaptonuria

Variable	TPO antibodies, median (IQR)		*P* value
	Positive	Negative	
Age at diagnosis of hypothyroidism, y	42 (17-57)	53 (47-58)	.10
Women, No./total No. (%)	6/9 (67)	8/11 (73)	.44
Serum tyrosine, mg/dL	5.02 (1.41-8.70)	1.14 (0.91-1.23)	.61
Urine HGA, mmol/mol Cr	1732 (1481-2967)	2044 (988-2865)	.95
Thyroid volume, mL	14 (13.6-14.2)	4 (3.7-5.6)	.01

Abbreviations: Cr, creatinine; HGA, homogentisic acid; IQR, interquartile range; TPO, thyroid peroxidase.

SI conversion factor: To convert tyrosine to micromoles per liter, multiply by 55.19.

Thyroid ultrasonographic scan data were available in 49 patients. Thyroid nodules were identified with a median size of 0.4 cm (IQR, 0.3-3.2 cm) in 29 patients (59.2%), which was within the known prevalence of 68% as observed in the general population (difference, 0.088; 95% CI, −0.44 to 0.73; *P* = .20).^18^ Three patients (6%) were diagnosed with thyroid cancer, 2 with micropapillary thyroid cancer and 1 with Hurthle cell thyroid cancer. The prevalence of thyroid cancer was similar to that in the general population (7% vs 5%; difference, 0.01; 95% CI, −0.01 to 0.17; *P* = .86).^18^

**Figure 2.**
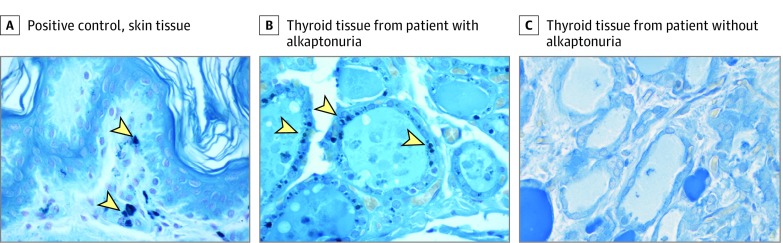
Pigment Deposition in Thyroid Tissue A, Positive control tissue sample. B, Thyroid tissue from a patient with alkaptonuria who underwent thyroidectomy. C, Thyroid tissue from a patient without alkaptonuria who underwent thyroidectomy. Arrowheads indicate pigment deposition.

We performed Schmorl staining on formalin-fixed, paraffin-embedded thyroid specimens from 4 individuals with alkaptonuria and 1 individual without alkaptonuria who underwent thyroidectomy. The staining revealed significant pigment accumulation in the follicular cells of the thyroid gland of individuals with alkaptonuria, suggesting that accumulation of HGA may play a role in thyroid gland disease (Figure 2).

## Discussion

To our knowledge, we report the largest US cohort of patients with alkaptonuria and suggest a significantly higher prevalence of primary hypothyroidism in adults with alkaptonuria compared with the general population.^10^ Most patients presented with overt hypothyroidism and were treated with a median levothyroxine dose of 1.4 μg/kg of body weight. The significance of diagnosis of subclinical hypothyroidism in 4.8% of the patients in this cohort is unclear and needs longitudinal follow-up, as some of these individuals may develop overt hypothyroidism over time. Consistent with the data observed in the general population of patients with hypothyroidism, female sex was associated with a higher likelihood of hypothyroidism compared with male sex, and the presence of TPO antibodies was associated with a higher likelihood of hypothyroidism compared with TPO antibody–negative status in patients with alkaptonuria.^12^

In line with previous case reports suggesting an association between alkaptonuria and autoimmune diseases,^19,20^ we hypothesized that there would be a higher prevalence of autoimmune thyroid disease in this population. We found that approximately 50% of patients with alkaptonuria and primary hypothyroidism were positive for TPO antibodies, which is significantly less than 82% of TPO antibody positivity seen in the general population with primary hypothyroidism.^17^ We found 2 causes for hypothyroidism in patients with alkaptonuria: 50.0% had typical autoimmune disease, characterized by female sex predominance and the presence of TPO antibodies, while the remaining half of the patients without TPO antibodies were characterized by slightly, not statistically significant, older age and significantly lower thyroid volume, suggesting a unique cause of hypothyroidism.

Although a subset of patients with alkaptonuria were transiently receiving nitisinone therapy, which blocks the first step in the tyrosine degradation pathway, the diagnosis of primary hypothyroidism in this subgroup preceded the initiation of nitisinone therapy, suggesting no association between hypothyroidism and exposure to nitisinone. There was also no association between primary hypothyroidism and serum tyrosine and urine HGA levels, which led us to test the tissue of 4 patients who underwent thyroidectomy. The Schmorl stain on thyroid tissue revealed significant pigment deposition in the thyrocytes (Figure 2). Accumulation of HGA in various tissues of individuals with alkaptonuria leads to severe degenerative abnormalities, including osteoarthropathy, mitral or aortic valve disease, and kidney or prostate stones.^1,5^ In light of these observations, we hypothesize that HGA accumulation in the thyroid gland may cause dysfunction of the gland over time, and functional in vitro and in vivo studies are needed to investigate a potential role of HGA in thyroid hormone synthesis by follicular cells (eFigure in the Supplement).

In this cohort, there was no significant difference in the prevalence of hyperthyroidism in patients with alkaptonuria compared with the general population. To date, there are few case reports on concomitant presentation of alkaptonuria and thyrotoxicosis.^21^ Our patients with alkaptonuria had a similar prevalence of thyroid nodules and cancer compared with the general population.^18^ However, the subset of patients who underwent screening for thyroid nodules and cancer with thyroid ultrasonographic scanning was relatively small; thus, further studies with larger sample sizes are needed to support our findings. To our knowledge, there have been no reports on the association of alkaptonuria with thyroid nodules or cancer.

### Strengths and Limitations

This study has strengths and limitations. The major strength of our study is that it analyzed thyroid function tests in, to our knowledge, the largest reported cohort of patients with alkaptonuria. Data on potential confounders affecting thyroid function tests, such as comorbid conditions, medications, and thyroid surgery, were identified. A limitation of this study is its retrospective design, which is associated with potential selection bias, as well as the lack of information on certain confounders, such as TPO antibodies in a subset of patients, which may affect the accuracy of the logistic regression model.

Most patients with overt hypothyroidism were diagnosed before their enrollment in this study; thus, thyrotropin and free thyroxine level values preceding levothyroxine therapy were not available for the analysis. However, the diagnosis of overt hypothyroidism in these patients is confirmed by the fact that they required levothyroxine therapy at a median of 1.4 μg/kg of body weight to maintain clinical and biochemical euthyroidism. Another limitation of our study is that it was based on a median of 3 thyroid function test measurements over time and compared with epidemiologic data based on a single thyrotropin measurement, potentially leading to an overestimation of a difference. Moreover, the reference population was characterized by a higher and potentially confounding prevalence of women, because female sex has been associated with hypothyroidism. Direct comparison of 2 demographically different populations is associated with an inherent error. However, it seems as though the lower proportion of women in this cohort and yet higher prevalence of hypothyroidism provides more support for the association between alkaptonuria and hypothyroidism. Although the design of our study enables description of an association between alkaptonuria and hypothyroidism, cohort studies are unable to determine causal inference.

## Conclusions

Our study has clinical implications, as the high prevalence of primary hypothyroidism noted in patients with alkaptonuria suggests that screening for primary hypothyroidism in this population should be considered. We believe that the first screening should occur at alkaptonuria diagnosis, as the youngest patient diagnosed with hypothyroidism in this cohort was aged 8 years, and should be repeated in regular intervals (eg, every 1-2 years) thereafter. The presence of primary hypothyroidism in patients without TPO antibodies suggests a potentially unique cause of hypothyroidism in this population and forms a basis for further research studies on a functional role of HGA deposition in the thyroid gland.

## Notes

Supplement. **eFigure.** The Crosstalk Between the Tyrosine Degradation Pathway and Thyroid Hormones Synthesis Pathway **eTable.** Serum Tyrosine and Urine HGA Concentration in Hypothyroid and Euthyroid Patients Treated With Nitisinone
