# Supplementary material for: Assessment of Thyroid Function in Patients With Alkaptonuria
Source: JAMA Netw Open. 2020 Mar 23;3(3):e201357. doi: 10.1001/jamanetworkopen.2020.1357 (PMC7090965; doi:10.1001/jamanetworkopen.2020.1357)
Supplement: Supplement. — eFigure. The Crosstalk Between the Tyrosine Degradation Pathway and Thyroid Hormones Synthesis Pathway eTable. Serum Tyrosine and Urine HGA Concentration in Hypothyroid and Euthyroid Patients Treated With Nitisinone [file jamanetwopen-3-e201357-s001.pdf]

Supplementary Online Content

Avadhanula S, Introne WJ, Auh S, et al. Assessment of thyroid function in patients with alkaptonuria. *JAMA Netw Open*. 2020;3(3):e201357.
doi:10.1001/jamanetworkopen.2020.1357

eFigure. The Crosstalk Between the Tyrosine Degradation Pathway and Thyroid Hormones Synthesis Pathway

eTable. Serum Tyrosine and Urine HGA Concentration in Hypothyroid and Euthyroid Patients Treated With Nitisinone

This supplementary material has been provided by the authors to give readers additional information about their work.

© 2020 Avadhanula S et al. *JAMA Network Open.*

eFigure. The crosstalk between the tyrosine degradation pathway and thyroid hormones synthesis pathway.

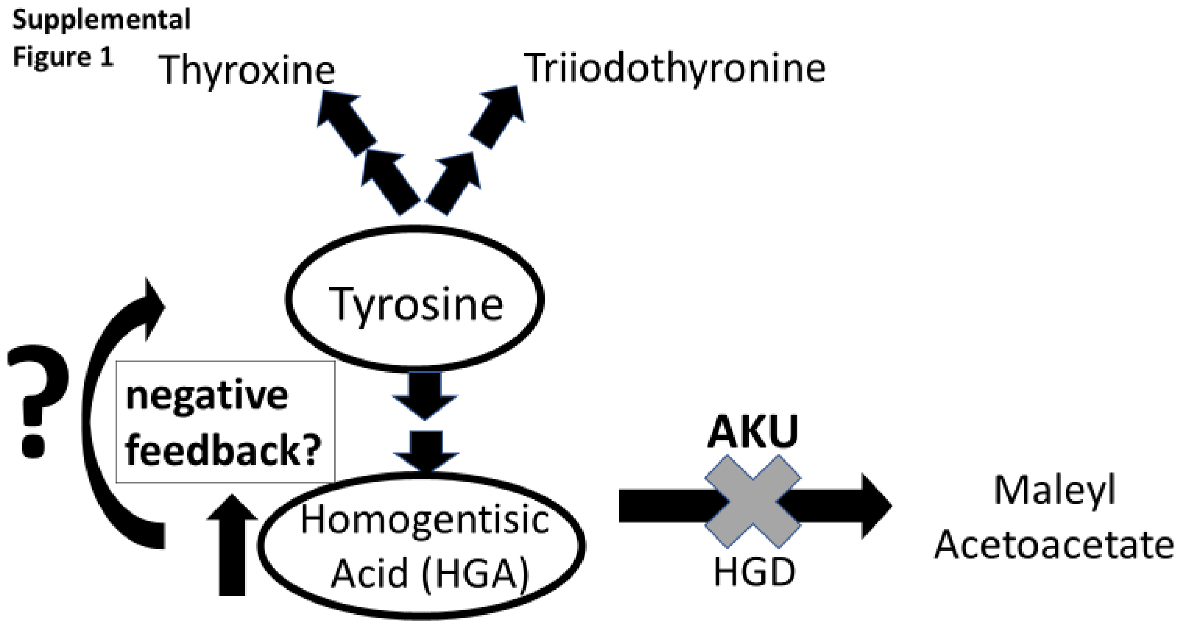

© 2020 Avadhanula S et al. *JAMA Network Open.*

eTable. Serum tyrosine and urine HGA concentration in hypothyroid and euthyroid patients treated with nitisinone.

	**Hypothyroid patients on nitisinone (n=4)***	**Euthyroid patients on nitisinone (n=13)**	**P value**
Median tyrosine nmol/ml [25-75%IQR]	619 [450.4-914.8]	750.5 [601.5-789]	0.98
Median urine HGA mmol/molCr [25-75% IQR]	114 [81.3-140.6]	84 [67.4-117.4]	0.61
	**Hypothyroid patients not treated with nitisinone (n=16)**	**Euthyroid patients not treated with nitisinone (n=90)**	
Median tyrosine nmol/ml [25-75%IQR]	56.5 [46.3-64.5]	54.5 [47.1-60.6]	0.9
Median urine HGA mmol/molCr [25-75% IQR]	2584.5 [1545.9-2962.7]	2439.7 [1501-3221.4]	0.67

*There were total 5 hypothyroid patients treated with nitisinone, but 1 patient was excluded due to post-thyroidectomy etiology of hypothyroidism

© 2020 Avadhanula S et al. *JAMA Network Open.*
